# Emotional Burden among Pharmacists and Pharmacy Technicians during the COVID-19 Lockdown: A Cross Sectional Study

**DOI:** 10.3390/ijerph191710558

**Published:** 2022-08-24

**Authors:** Carmen Baldonedo-Mosteiro, María-Pilar Mosteiro-Díaz, Sara Franco-Correia, Adonina Tardón

**Affiliations:** 1 Centro Internacional de Postgrado, Universidad de Oviedo, 33003 Oviedo, Spain; 2 Grupo de Investigación INEUROPA, Departamento de Medicina, Área de Enfermería, Universidad de Oviedo, 33006 Oviedo, Spain; 3 Departamento de Medicina, Área de Enfermería, Universidad de Oviedo, 33006 Oviedo, Spain; 4 Departamento de Medicina, Área de Salud Pública, Health Research Institute of Investigation (ISPA) and CIBERESP, Universidad de Oviedo, 33006 Oviedo, Spain

**Keywords:** anxiety, depression, pharmacist, pharmacy technician, community, COVID-19

## Abstract

This study aims to investigate the prevalence of depression and anxiety symptoms among Spanish community pharmacists and pharmacy technicians during the coronavirus disease 2019 (COVID-19) lockdown. A descriptive cross-sectional quantitative study was designed. An online survey was administered to participants from 4 to 21 April 2020 using a questionnaire assessing sociodemographic information and the Spanish version of the Hospital Anxiety and Depression Scale (HADS). Informed consent to participate was requested. Participants comprised 1162 pharmacy staff from Spain with an average age of 39.15 ± 9.718, from 20 to 65 years old, of whom 83% were women, and 50.6% were married. More than half of the participants expressed symptoms of depression (62.7%) and anxiety (70.9%). An important prevalence of anxiety and depression symptoms has been detected among Spanish pharmacists and pharmacy technicians during the COVID-19 lockdown. Being a woman, smoking, feeling fear, feeling stress and believing that pharmacists/pharmacy technicians are very exposed to COVID-19 seem to be associated with higher HADS scores.

## 1. Introduction

The novel coronavirus disease 2019 (COVID-19) caused by severe acute respiratory syndrome coronavirus 2 (SARS-CoV-2) was declared a pandemic on 11 March 2020 by the World Health Organization [1]. As of 30 April 2020, and since the beginning of the COVID-19 pandemic, the Spanish Government reported 212,917 confirmed cases of COVID-19 and 24,275 deaths [2]. It was at this moment when Spain was experiencing the highest number of cases, and Italy was recording the highest number of deaths [3].

In an attempt to reduce the spread of the virus, countries around the world were enforcing lockdowns. The Spanish Government declared the official national lockdown on 14 March 2020 [4]. Pharmacies in many countries at a national level are considered essential services and are one of the few amenities that remained open and accessible to the public when countries were placed into lockdown [5]. Specific recommendations have been published regarding the continued operation of professional pharmacy services in community pharmacies, which include adapting their facilities with security measures, social distancing, the use of personal protective equipment (PPE) and methods to decrease contact with objects handled by patients. The use of alternative prescription delivery methods was also encouraged, including pharmaceutical home care by delivering directly to patients’ homes in order to limit direct patient contact in the pharmacy [6,7]. The need to prevent the spread of COVID-19 transmission has induced changes in the form and nature of performing medication reconciliation, conducting patient interviews and providing health education [8,9]. During this period, healthcare workers (HCWs) have faced many changes regarding their practices, safety protocols and policies, with specific modifications to workflow including the use of PPE [10].

A wide range of healthcare services are provided by pharmacy staff in community pharmacies—healthcare hubs designed to meet the health needs of communities [11,12]. Since the beginning of the COVID-19 outbreak, pharmacists worldwide have been playing a key role in adopting innovative strategies to minimize the adverse impact of the pandemic. Such professionals have been dedicating their activity not only to reducing the possibility of virus spread but also to patients’ daily needs related to taking medicines [13].

HCWs feel a dominant threat of being exposed and infected as a result of continuing to work in fearful, stressful, and constrained settings [14]. Due to prolonged work shifts, scarcity of PPE, and fear of being infected as well as infecting their families as a possible infecting vehicle generates an increased daily pressure on these professionals [15]. Indeed, their physical and mental well-being are being affected [16].

Mental health is an important component of public health, especially in times of crisis [17]. Negative outcomes of the COVID-19 pandemic on people’s mental health have been described in a wide range of studies [18]. It is also claimed that some groups may be more vulnerable than others to the psychosocial effects of pandemics with an increased risk for adverse psychosocial outcomes: people who contract the disease, those at heightened risk for it, people with preexisting medical, psychiatric, or substance use problems, and HCWs [19,20]. Exacerbation of pre-existing mental illness, increased stress levels, anxiety, and depressive symptoms, are described as a result of the conditions associated with the pandemic situation [21,22,23,24].

Negative psychological effects of COVID-19 on HCWs worldwide have been reported including stress, fear, anxiety, depression, burnout, sleep disorders, and mental exhaustion [24,25,26,27]. Numerous clinical outcomes, including stress, depression, irritability, insomnia, fear, confusion, anger, frustration, boredom, and stigma associated with quarantine, were identified in a recent review of psychological sequelae in samples of quarantined people and of HCWs [21]. Pharmacists and pharmacy technicians have been affected early by the pandemic situation. Given their risk of exposure to the virus in the current pandemic, frontline HCWs—pharmacists and pharmacy technicians included—are particularly vulnerable to emotional distress and to develop psychiatric disorders due to concerns about infecting and caring for their loved ones, longer working hours, and coping with stressful community events [28,29].

Due to the mental effects of the pandemic on HCWs, their attention, understanding decision-making capacity and wellbeing might be affected [30].

Pharmacists and pharmacy technicians are HCWs who might feel that their lives are especially affected by the current COVID-19 pandemic and associated lockdown. In fact, many studies have reported that HCWs are experiencing significant psychological morbidity. However, pharmacists are not yet well represented in this research topic, and it is crucial to understand the impact of COVID-19 on pharmacists [31].

As shown, the mental health of HCWs during the COVID-19 pandemic and lockdown periods needs to be addressed. Pharmacists and pharmacy technicians face many stressors that need to be specifically targeted to address their mental health issues adequately. We believe that research is needed to explore the effects of COVID-19 on mental health and psychological wellbeing among this population and to identify the factors that contribute to these effects.

The main objective of the study was to describe the prevalence and levels of anxiety and depression symptoms among Spanish community pharmacists and pharmacy technicians during the COVID-19 outbreak lockdown and to examine factors contributing to high levels on the Hospital and Anxiety Depression Scale (HADS).

## 2. Materials and Methods

### 2.1. Study Design

This study used a quantitative, correlational and cross-sectional design through non-probabilistic sampling using the “snowball” method. An online questionnaire was created using a Google Form; the link was also made available on social media. Data were collected from 4 to 21 April 2020, covering most of the Spanish regions.

### 2.2. Participants

Pharmacists and pharmacy technicians across the whole of Spain were enrolled. The inclusion criterion was to be a community pharmacist or pharmacy technician. Exclusion criteria included forms sent after 21 April 2020 and any forms with incomplete data. A total of 1162 participants with an average age of 39.15 years (SD = 9.718) were included in this study and there were no missing data.

### 2.3. Measures

An online questionnaire was applied consisting of questions organized as follows: questions addressing sociodemographic data (age, gender, region, pharmacy location professional category, marital status, children, educational level, local incidence) and “yes/no” questions about general aspects regarding COVID-19 and psychological symptoms: “Do you think pharmacists/pharmacy technicians are very exposed to the COVID-19 at work?”, “Do you feel fear?”, “Do you feel stress?”, “Do you have any infected close family members?”, “Do you have any infected friends?”, “Did you have any symptoms?”, and “Do you live with anyone who has been infected?”. Sociodemographic information and “yes/no” questions were asked to participants before the HADS. This part takes less than 5 min to be answered.

The Hospital Anxiety and Depression Scale (HADS) was used to assess the psychological symptoms. We used the version that had been adapted and validated for the Spanish population [32]. The HADS comprises 14 multiple-choice questions divided into two subscales measuring symptoms of depression (HADS-D) and anxiety (HADS-A). Seven questions are asked in each category about symptoms experienced over the past week, scored on a four-point scale. Scores for each element varied from 0 to 3: Did not apply to me at all = 0; Applied to me to some degree or some of the time = 1; Applied to me to a considerable degree or a good part of the time = 2; Applied to me very much or most of the time = 3. The overall score for each subscale, when added up independently for anxiety and depression, ranges from 0 to 21. A higher total score depicts a worse condition [33].

According to Herrero et al., there is no single, generally accepted cut-off score for the HADS [32]. In the original study, two cut-off scores were recommended for both subscales: 7/8 for possible and 10/11 for probable anxiety or depression (with a possible range of 0–21 for each subscale); a third cut-off score of 14/15 for severe disorder was also proposed [34,35]. Another study reveals that the optimal cut-off score for the HADS was ≥ 9, with HADS-D ≥ 4 [36]. A score of ≥ 11 is considered to be a clinically significant disorder, whereas a score of 8–10 suggests a mild disorder [34]. Other study experience enabled each mood state to be divided into four ranges—normal (0–7), mild (8–10), moderate (11–14) and severe (15–21)—and it is in this form that the HADS is now issued [37] and adopted in our study for both HADS-A and HADS-D.

The HADS was preferred because it is easy to understand, fast to apply and includes few elements. It takes approximately 5 min to fill in and can be evaluated quickly [33]. A study showed that the internal consistency (Cronbach’s alpha) was found to be 0.81 for HADS-A, 0.71 for HADS-D and 0.85 for HADS total [38]. The validation study of the HADS Spanish version indicates a Cronbach’s alpha of 0.90 for HADS total, 0.84 for HADS-D and 0.85 for HADS-A [32]. In another study, the HADS test-retest reliability presented correlation coefficients above 0.85. The internal consistency was high, with Cronbach’s α of 0.86 for HADS-A and 0.86 for HADS-D [39].

### 2.4. Data Analyses

A brief descriptive analysis was performed on each collected variable, with position measures (e.g., mean, minimal, maximal and median) or dispersion measures (e.g., standard deviation) for quantitative variables and distribution measures of absolute and relative frequencies for qualitative variables.

Differences between quantitative variables of two groups were assessed with Student’s *t*-test for independent normal samples. For groups of three or more, analysis of variance (ANOVA) or the Kruskal-Wallis test were used, according to normality or homoscedasticity. Multivariate models were built for anxiety and depression. Shapiro-Wilk normality test was used to analyze the sample distribution. The level of statistical significance was defined as 0.05. Statistical analysis was performed using SPSS Inc., v.22. Chicago, IL, USA.

### 2.5. Ethical Aspects

The first part of the e-form consisted of general information and the aim of the research. Informed consent was obtained from all participants included in the study. After reading the Informed Consent, individuals accepted it by clicking on the button that configured the voluntary participation agreement in the study. Confidentiality and anonymity were ensured.

This study was performed in line with the principles of the Declaration of Helsinki. Approval was obtained from the Ethical Committee of Research of the Principality of Asturias (CeiPA2020.116).

## 3. Results

### 3.1. Sample Characteristics

The sample was made up of 1162 participants who completed the survey. More than half (63.6%) were pharmacists, with a mean age of 39.15 ± 9.718, from 20 to 65 years old. Most were female (86.7%), married (67.2%), with no children (50.6%), non-smokers (81.4%), from the north region of Spain (45.2%), working in large city pharmacies (40.1%) and with a local COVID-19 incidence of 10,001–15,000 (27.5%). The majority (94.0%) thought that pharmacists/pharmacy technicians are very exposed to COVID-19. Within the participants, more than half self-reported feeling fear (55.9%) and a larger number reported feeling stress (90.4%) (Table 1).

### 3.2. HADS: Anxiety and Depression Symptoms Prevalence and Levels

Regarding the HADS scores, five participants did not fully complete the questionnaire and the data were excluded from analysis. Regarding to anxiety levels among the sample during the COVID-19 lockdown, 70.9% presented some level of anxiety disorder (HADS-A ≥ 8) and quartile analysis indicated that 50% revealed HADS-A scores above 10.00. With respect to depression levels among the sample, 62.7% revealed anxiety symptoms, 29% presented significant depression (HADS-D > 11) (Table 2); quartile analysis indicated that half of the participants had HADS-D scores below 9.00.

### 3.3. Correlation Analyses

#### 3.3.1. HADS-D Correlation Analysis

Statistically significant differences were found (*p* < 0.05) between HADS-D levels and the following variables: gender; “Do you think that pharmacists/pharmacy technicians are very exposed to the COVID-19 at work?”; smoking habits; “Do you feel fear?”; “Do you feel stress?”; “Did you have any symptoms?”; and “Do you have any infected friends?”. For the remaining variables there was no significant association. Table 3 summarizes the statistically significant associations found.

While using the ANOVA test to describe the possible HADS-D model with defined factors, we observed that this model explains 20.3% of data variance (Table 4).

#### 3.3.2. HADS-A Correlation Analysis

Searching for an association between HADS-A and the remain variables, statistically significant difference were found with the following: gender; local incidence; “Do you think that pharmacists are very exposed to the COVID-19 at work?”; smoking habits; “Do you feel fear?”; “Do you feel stress?”; “Did you have any symptoms?”; and “Do you have any infected friends?”. Table 5 summarizes the statistically significant associations.

The same analysis as performed before was made in order to present the factors contributing to HADS -D model. We observed that this model explains 25.2% of the data variance (Table 6).

## 4. Discussion

Pharmacists are considered frontline HCWs as they have been providing services that proved to be especially helpful and necessary during the COVID-19 pandemic [7]. Therefore, they have been facing many stressors that must be identified for the purpose of addressing mental health issues. Our study aimed to describe the prevalence of symptoms of anxiety and depression among Spanish community pharmacists and pharmacy technicians during the COVID-19 outbreak and to identify key factors that contribute to higher anxiety and depression levels.

In relation to the prevalence of symptoms of anxiety and depression, our findings indicate that 70.9% of the participants presented anxiety (scores in HADS-A ≥ 8) and 62.7% revealed symptoms of depression. The findings of a study on 803 HCWs from Bangladesh, using HADS, indicate lower anxiety and depression prevalence, with 69.5%, and 39.5%, respectively [40]. Lower incidence was also found in another research study. A study performed on HCWs in Singapore, but not using the same tool, revealed that the non-medical group, which included pharmacists and pharmacy technicians, had a prevalence of 20.7% for anxiety and 10.3% for depression [41].

Regarding HADS-A levels, 34.8% and 9.6% revealed moderate and severe anxiety symptoms, respectively. A very different prevalence was found in the study by Özdin and Bayrak Özdin [42]: 23.6% (*n* = 81) for HADS-D and 45.1% (*n* = 155) HADS-A cut-off point. Lower incidences were also found in an Italian study of 2195 HCWs, where 50.1% reported symptoms of clinically significant anxiety and 26.6% reported symptoms of at least moderate depression [43]. In a cross-sectional study of 2638 HCWs in the UK, mental health symptoms were detected at a prevalence of 34.3% for significant anxiety and 31.2% for significant depression [44]. According to the large number of studies found, the outbreak of COVID-19 was probably a trigger to focus on the mental health among HCWs. Nonetheless, it is important to highlight that all of these studies have been performed during the current COVID-19 pandemic and few have been dedicated to pharmacists or pharmacy technicians. They reflect the real picture of mental health among populations on a specific moment. Despite the fact that it would have been interesting to compare anxiety and depression prevalence and levels obtained to pre-COVID era, there is no available data.

Regarding the association between certain characteristics of the participants and the prevalence of higher anxiety symptoms, our results indicate that the following can be predictors: being female, smoking, believing that pharmacists and pharmacy technicians are very exposed to COVID-19 at work, local incidence of 10,000 to 15,000, feeling fear, and feeling stress. While it would seem an interesting feature, no data to compare or to help us to understand it or explain these results were found.

The regression analysis indicates that “Do you feel fear)”, “Do you feel stress?”, “Did you have any symptoms?”, “Do you have any infected close family members?”, and “Do you have any infected friends?” might be predictors to high HADS-A score.

The characteristics that seem to be associated with a higher risk of depression are being female, smoking, believing that pharmacists are very exposed to COVID-19 at work, feeling fear and feeling stress. On a study performed in Italy on 1379 HCWs during the first Italian lockdown, female gender was associated with higher depression level (*p* < 0.001) [45]. Overall, studies performed during the pandemic indicate that women may be at higher risk of developing anxiety and depression symptoms than men [46,47,48]. Limited literature is available to allow any discussion or comparison of such results. Being female and smoking were associated with higher levels of anxiety and depression. In a systematic review, the goal of which was to describe the association of smoking with anxiety and depression, inconsistent findings were found. In fact, across the multiple analyzed studies, authors detected that depression and anxiety also leads to smoking or increased smoking behavior. In terms of the direction of association, the association between anxiety and depression with smoking is inconsistent [48]. Indeed, female gender has been associated with worse mental health outcomes during this pandemic period which implies major psychological impact levels. In a systematic review of mental health in HCWs during the pandemic, it was found that being a woman was one of the most common risk factors for mental health problems in HCWs [49]. A study analyzing the psychological impact on this population group revealed that being female was associated with higher Impact Event Scale (IES-R) mean scores [50].

In this study, some limitations to the interpretation of results have been identified that must be taken into account. As a voluntary sample recruited online within a short time period, it was not possible to randomize the study. Moreover, the participants were not asked about the existence of previous mental disorders. We are aware that a clearer picture of the psychological impact in the face of the pandemic would be possible if such aspects were considered.

This evidence suggests the existence of a group with greater vulnerability for high-stress situations that requires greater attention in terms of proposals for its management. Screening for mental health problems, psychoeducation and psychosocial support should focus on these and other groups at risk of adverse psychosocial outcomes and take preventative measures. Consequently, a widespread psychiatric care service is needed, as well as expanded training of more qualified professionals in the management of the psychological impact of the pandemic, in order to reduce mental health problems in this specific population. More focus and developed resources are needed to provide solutions to promote psychological well-being of pharmacists, pharmacy technicians and HCWs in general amid COVID-19.

It is also recommended to look into burnout screening and prevention strategies in this professional group and the use of the following measures is suggested: employee assistance, counseling, organization workflow and standardization, mindfulness, organizational screening.

## 5. Conclusions

Pharmacists and pharmacy technicians saw their mental health affected by the COVID-19 outbreak, revealing a high prevalence of symptoms of anxiety and depression of 70.9% and 62.7%, respectively. Being female, smoking, feeling fear, feeling stress and believing that pharmacists are very exposed to COVID-19 at work have all been associated with a higher risk for developing anxiety and depression.

There is an urgent need to deliver integrated and stronger policies to support mental health. Such policies should include promoting the access to existing mental health services either via telemedicine or in-person.

## Figures and Tables

**Table 1:** Participants’ characteristics.

Variable	Type	N	%
Gender	Female Male	1008	86.7
		154	13.3
Professional category	Pharmacist Pharmacy technician	739	63.6
		423	36.4
Marital status	Single Married/living with partner Divorced/separated Widow	312	26.9
		781	67.2
		61	5.2
		8	0.7
Region of Spain	North Central South Islands Not recorded	525	45.2
		386	33.2
		167	14.6
		68	5.9
		16	1.4
Local incidence	<3000 3000 to 5000 5001 to 10,000 10,001 to 15,000 15,001 to 20,000 >20,000	295	25.4
		64	5.5
		90	7.7
		320	27.5
		130	11.2
		263	22.6
Pharmacy location	Town Small city Large city	362	31.2
		334	28.7
		466	40.1
Smoking habits	No Yes	946	81.4
		216	18.6
Children	No Yes	588	50.6
		574	49.4
Do you think pharmacists/pharmacy technicians are very exposed to the COVID-19 at work?	No Yes	70	6.0
		1092	94.0
Do you feel fear?	No Yes	513	44.1
		649	55.9
Do you feel stress?	No Yes	111	9.6
		1051	90.4
Do you have any infected close family members?	No Yes	991	85.3
		171	14.7
Do you have any infected friends?	No Yes	709	61.0
		453	39.0
Do you live with anyone who has been infected?	No Yes	1106	95.2
		56	4.8

**Table 2:** The Hospital Anxiety and Depression Scale scores and levels.

	Anxiety			Depression		
Mean	9.64			8.68		
SD	3.882			3.224		
Quantiles	1	2	3	1	2	3
	7.00	10.00	12.00	6.00	9.00	11.00
Levels	n		%	n		%
Normal (0–7)	333		28.7	429		36.9
Mild (8–10)	308		26.5	391		33.7
Moderate (11–14)	404		34.8	290		25.0
Severe (15–21)	112		9.6	47		4.0
Not analyzed	5		0.4	5		0.4

**Table 3:** Correlation analysis of the HADS-D levels.

	Normal	Mild	Moderate	Severe	p *
	Gender				
Female	336	353	273	41	0.000
Male	93	38	17	6	
	Do you think that pharmacists/pharmacy technicians are very exposed to the COVID-19 at work?				
No	43	13	11	3	0.000
Yes	386	378	279	44	
	Smoking habits				
No	374	304	228	36	0.000
Yes	55	87	62	11	
	Do you feel fear?				
No	266	153	86	5	0.000
Yes	163	238	204	42	
	Do you feel stress?				
No	86	22	2	1	0.000
Yes	343	369	288	46	
	Did you have any symptoms?				
No	345	299	203	28	0.001
Yes	84	92	87	19	
	Do you have any infected friends?				
No	290	242	148	29	0.000
Yes	139	149	142	18	

* Chi^2^ test.

**Table 4:** Predicting HADS-D model.

	Type III Square Sum	gl	Mean Square	F	Sig.
Corrected model	2558.373 ^a^	15	170.558	20.574	0.000
Interception	1605.842	1	1605.842	193.712	0.000
Gender	98.742	1	98.742	11.911	0.001
Marital status	50.139	3	16.713	2.016	0.110
Children	4.451	1	4.451	0.537	0.464
Professional category	9.656	1	9.656	1.165	0.281
Smoking habits	42.454	3	14.151	1.707	0.164
Do you feel fear?	675.084	1	675.084	81.435	0.000
Do you feel stress?	549.965	1	549.965	66.342	0.000
Did you have any symptoms?	100.397	1	100.397	12.111	0.001
Do you have any infected close family members?	12.365	1	12.365	1.492	0.222
Do you have any infected friends?	112.810	1	112.810	13.608	0.000
Age	61.277	1	61.277	7.392	0.007

^a^ R^2^ = 0.213 (R^2^ Adjusted = 0.203).

**Table 5:** Correlation analysis of HADS-A levels.

	Normal	Mild	Moderate	Severe	*p*
	Gender				
Female	267	275	357	104	0.000 *
Male	66	33	47	8	
	Local incidence				
<3000	113	71	87	23	0.008
3000 to 5000	21	19	18	5	
5001 to 10,000	22	22	32	13	
10,001 to15,000	80	96	119	25	
15,001 to 20,000	33	32	50	15	
>20,000	64	68	98	32	
	Do you think that pharmacists/pharmacy technicians are very exposed to the COVID-19 at work?				
No	38	15	15	2	0.000 *
Yes	295	293	389	110	
	Smoking habits				
No	289	242	324	87	0.000 *
Yes	44	66	80	25	
	Do you feel fear?				
No	237	120	136	17	0.000 *
Yes	96	188	268	95	
	Do you feel stress?				
No	86	14	11	0	0.000 *
Yes	247	294	393	112	
	Did you have any symptoms?				
No	273	237	292	73	0.001 *
Yes	60	71	112	39	
	Do you have any infected friends?				
No	215	211	226	57	0.000 *
Yes	118	97	178	55	

* Chi^2^ test.

**Table 6:** Predicting HADS-A model.

	Type III Square Sum	gl	Mean Square	F	Sig.
Corrected model	4562.668 ^a^	15	304.178	26.999	0.000
Interception	1701.082	1	1701.082	150.988	0.000
Gender	17.687	1	17.687	1.570	0.210
Marital status	32.253	3	10.751	0.954	0.414
Children	0.142	1	0.142	0.013	0.910
Professional category	0.228	1	0.228	0.020	0.887
Smoking habits	23.070	3	7.690	0.683	0.563
Do you feel fear?	1502.304	1	1502.304	133.344	0.000
Do you feel stress?	1312.596	1	1312.596	116.506	0.000
Did you have any symptoms?	136.050	1	136.050	12.076	0.001
Do you have any infected close family members?	20.572	1	20.572	1.826	0.177
Do you have any infected friends?	86.475	1	86.475	7.676	0.006
Age	36.507	1	36.507	3.240	0.072

^a^ R^2^ = 0.262 (R^2^ Adjusted = 0.252).

## Data Availability

Data sharing is not applicable to this article.
